# Insensitivity to T790M mutation? A pooled analysis of outcomes following osimertinib for the treatment of NSCLC patients harboring uncommon epidermal growth factor receptor mutation

**DOI:** 10.3389/fphar.2022.986962

**Published:** 2022-08-26

**Authors:** Shanliang Hu, Congjie Wang, Chunsheng Wang, Kewei Zhao, Zhen Wang, Wei Dong

**Affiliations:** ^1^ Department of Radiation Oncology, Yantai Yuhuangding Hospital, Yantai, China; ^2^ Department of Pulmonary and Critical Care Medicine, Yantai Yuhuangding Hospital, Yantai, China; ^3^ Department of Radiation & Medical Oncology, Zhongnan Hospital of Wuhan University, Wuhan, China; ^4^ Cancer Center, Union Hospital, Tongji Medical College, Huazhong University of Science and Technology, Wuhan, China

**Keywords:** osimertinib, uncommon, EGFR, Efficacy, Prognosis, NSCLC6

## Abstract

**Background:** In this pooled analysis, the aim was to investigate the clinicopathological characteristics of patients with uncommon epidermal growth factor receptor (EGFR) (ucm-EGFRms) along with their treatment responses and survival following osimertinib treatment.

**Methods:** Univariate chi-square analysis was conducted to analyze the correlation between clinical characteristics, EGFR mutation type, and treatment response, and the Kaplan-Meier method was applied for survival analysis. Univariate logistic regression model and Cox proportional hazards model were performed to compare the efficacy and prognosis in subgroup analysis.

**Results:** Seventy-two NSCLC patients in total were included in this pooled analysis. The objective response rate (ORR) for osimertinib treatment was 57.0%, with a median PFS of 7.1 months. Twenty-eight patients received osimertinib as first-line therapy with an ORR of 67.9%, which was higher than that in patients who received osimertinib as second- or later-line therapy, and their response rate was 50%, nevertheless, no statistically significant differences were found (*p* = 0.139). However, patients who received first-line osimertinib showed a more significant PFS benefit than those who received second- or later-line therapy (mPFS: 16.8 months vs 6.0 months HR: 2.453, 95%CI: 1.285-4.682, *p* =0.004). Subgroup analysis showed that patients with a single, non-ex20ins, ucm-EGFRm displayed a superior efficacy advantage and favorable survival benefit following osimertinib treatment, with an ORR of 68.8% and an mPFS at 15.1 months. By contrast, patients with a multiple ucm-EGFRm that contain T790M exhibited the worst outcome of osimertinib treatment, with an ORR of 47.6% and an mPFS of only 3.6 months, respectively.

**Conclusion:** Patients with um-EGFRms exhibit favorable but inconsistent responses and survival outcomes following osimertinib treatment, which is closely related to the mutation pattern and cooccurring partner mutant genes. Administering osimertinib for the treatment of patients with um-EGFRm might be considered an effective treatment option in some circumstances.

## 1 Introduction

Epidermal growth factor receptor (EGFR) mutation is one of the main oncogenic drivers of non-small-cell lung cancer (NSCLC) (Rosell et al., 2009; D'Angelo et al., 2011). Deletion of exon 19 (19del) and point mutations of L858R in exon 21 represent approximately 80%–90% of EGFR mutations and are considered to be common and sensitive mutations of the EGFR (Rosell et al., 2009; D'Angelo et al., 2011; Sharma et al., 2007). Numerous clinical trials have proved that, compared to conventional platinum-based chemotherapy, epidermal growth factor receptor-tyrosine kinase inhibitors (EGFR-TKIs) produce better objective response rates (ORR) and progression-free survival (PFS), as well as overall survival (OS) (Mitsudomi et al., 2010; Zhou et al., 2011; Wu et al., 2014; Yang et al., 2014; Paz-Ares et al., 2017; Soria et al., 2018; Ramalingam et al., 2020). Additionally, other types of EGFR mutations, called uncommon EGFR mutations (ucm-EGFRms), were also been found, and account for approximately 10%–15% of EGFR mutations (Russo et al., 2019; John et al., 2022). Owing to the very small sample size and a high degree of heterogeneity, the effectiveness of EGFR-TKIs in patients having um-EGFRms remains uncertain (Russo et al., 2019; Zhang et al., 2020; John et al., 2022).

Osimertinib is the first third-generation EGFR-TKI that was approved by the Food and Drug Administration (FDA) as a treatment for advanced NSCLC patients who harbor common EGFR mutations (Cross et al., 2014; Mok et al., 2017). Currently, data concerning the clinical effectiveness of osimertinib in NSCLC patients harboring ucm-EGFRms are still limited. Hence, we performed this pooled analysis to investigate the clinical effectiveness and prognosis of applying osimertinib in NSCLC patients with ucm-EGFRms, so as to provide a reference for clinicians to formulate individualized and precise treatment plans for patients with ucm-EGFRms, especially in special populations.

## 2 Methods

### 2.1 Search strategy and study eligibility

An online literature search was conducted in the NCBI PubMed database to identify relevant articles. The following keywords were used: uncommon, mutation, osimertinib, AZD9291, EGFR, and NSCLC (the detailed search strategy is shown in the supplemental material). The titles and abstracts of the searched relevant articles were screened independently by two authors, and a second screening of the full-text articles was also conducted. Inclusion criteria were: 1) prospective and retrospective research, case or case series reports, as well as letters to the editor that focused on patients with EGFR mutation; 2) studies in which patients carried uncommon mutations in EGFR and received osimertinib in any treatment line; 3) studies with data on treatment outcomes.

### 2.2 Data extraction

Data for each study were collected on age, gender, smoking history, tumor stage, mutation type, brain metastasis state before osimertinib therapy, and treatment outcomes. Response to osimertinib, also known as a complete response, partial response, stable or progressive disease. Complete response and partial response were defined as the objective response (OR). The clinical outcomes include ORR and PFS.

### 2.3 Exploratory analysis

EGFR exon 20 insertions (ex20ins) mutations have low sensitivity to first- and second-generation EGFR-TKIs and the Food and Drug Administration (FDA) has approved amivantamab as a therapy for patients who have an EGFR ex20in mutations. We have great interest in the outcomes of osimertinib for those patients. In addition, osimertinib works well in patients with T790M mutations, but its effectiveness for patients with a combination of ucm-EGFRms is unclear. Therefore, we conducted an exploratory subgroup analysis of treatment response and survival for different ucm-EGFRms patterns: Group A for exon 20ins mutations, Group B for other single ucm-EGFRms, Group C for multiple ucm-EGFRms that contain T790M mutations, and Group D for multiple ucm-EGFRms that without containing T790 M mutations.

### 2.4 Statistical analysis

Comparisons of efficacy were conducted by chi-square analysis, with odds ratios (ORa) and 95% confidence intervals (CI) calculated using a logistic regression model. A Kaplan-Meier technique (log-rank tests) was used to estimate the PFS, while a Cox proportional hazards model was used to compute the hazard ratio (HR) and 95 %CI. Univariate logistic regression model and Cox proportional hazards model were performed to compare the efficacy and prognosis of different mutation subgroups, with ORas and HRs as well as 95% CI were calculated, respectively. Statistical significance was determined by a *p*-value less than 0.05 for two-sided tests. SPSS 23.0 program (SPSS Inc., Chicago, IL, United States) was used for the analysis.

## 3 Results

### 3.1 Search results

From the PubMed database, 397 potentially relevant articles were identified. Three hundred and seventy-four records were left after removing duplicates. By reading the titles and abstracts of the articles, 154 were excluded, including 59 non-clinical researches as well as 95 articles unrelated to osimertinib. The full texts of the remaining 220 articles were reviewed. An additional 181 articles were excluded since 69 focused on common EGFR mutations and 112 did not provide any efficacy or PFS data. Ultimately, 39 articles fulfilled the inclusion criteria and an additional five articles were determined by searching the reference lists of these full texts. Overall, forty-four articles were selected for inclusion in this pooled analysis (Supplementary Figure S1). The articles included in this research are presented in Supplementary Table 1.

### 3.2 Patient characteristics and epidermal growth factor receptor mutation analysis

Seventy-two patients were enrolled in this pooled analysis, at a median age of 61 years and an age range of 25–83 years, of whom 44 (61.1%) were female and 28 (38.9%) were male; the majority of them were at stage IV (68,94.4%), and approximately half of them (37,51.4%) had brain metastases before OSI treatment; 32 patients carried a single mutation, while the other 40 patients carried multiple mutations (Table 1). In these 72 patients, there were 44 kinds of ucm-EGFRm types. As shown in Supplementary Figure S2, the highest frequency was 20ins, with 16 cases, accounting for 22.2%, followed by 19 del variants, with 9 cases (12.5%), L858R/T854A, with 3 cases (4.2%), L747P, with 2 cases (2.8%), L858R/T790L, with 2 cases (2.8%), and L858R/T790M/L833V, with 2 cases (2.8%). The remaining mutation types all occurred in one case, each accounting for 1.4%, and these types included mutations such as G719S, H733L, S768I, G719X/S768I, and H773L/V774M.

**TABLE 1:** Baseline characteristics.

Characteristics	No. (*n* = 72)	Percentage (%)
Age		
<65	45	62.5
≥65	27	37.5
Gender		
Male	28	38.9
Female	44	61.1
Smoking		
Yes	23	31.9
No	41	56.9
NA	8	11.1
Stage		
I-III	4	5.6
Ⅳ	68	94.4
BM before OSI		
Yes	37	51.4
No	31	43.1
N/A	4	5.6
Mutation number		
Single	32	44.4
Multiple	40	55.6
OSI lines		
1 L	28	38.9
≥2 L	44	61.1
Response to TKI		
CR	2	2.8
PR	39	54.2
SD	18	25.0
PD	13	18.1
PFS		
median	7.1 m	-

NA, not available; BM, brain metastases; OSI, osimertinib; L: line.

### 3.3 Efficacy and PFS

**FIGURE 1:**
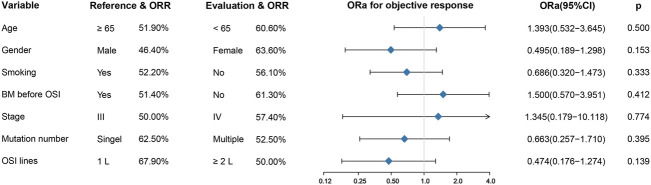
Univariate analysis for treatment response. There was no correlation between efficacy and age, gender, smoking history, brain metastatic state, tumor stage, mutation number, and OSI lines. BM: brain metastases; OSI: osimertinib.

In terms of the efficacy of osimertinib, of these seventy-two patients, two experienced a complete response (2.8%), thirty-nine experienced a partial response (54.2%), and eighteen experienced stable disease (25.0%), and thirteen experienced disease progression (18.1%). (Table 1). Altogether, the ORR for osimertinib treatment was 57.0% in all cohort patients. Twenty-eight patients who received osimertinib as first-line therapy had an ORR of 67.9%, which was higher than that of the remaining 44 patients who received osimertinib as second- or later-line therapy, and their response rate was 50%. Nevertheless, the difference between them was not statistically significant (ORa: 0.474, 95% CI: 0.176–1.274, *p* =0.139). In addition, patients who harbored a single ucm-EGFRm had an ORR of 62.5%, showing a slight favorable advantage over patients with multiple ucm-EGFRms, with an ORR of 52.5%; however, there were still no significant differences (ORa: 0.663, 95% CI: 0.257–1.710, *p* =0.395). Furthermore, there was no correlation between efficacy and age (*p* =0.500), gender (*p* =0.153), smoking history (*p* =0.333), brain metastatic state (*p* =0.412), or tumor stage (*p* =0.774). (Figure 1).

**FIGURE 2:**
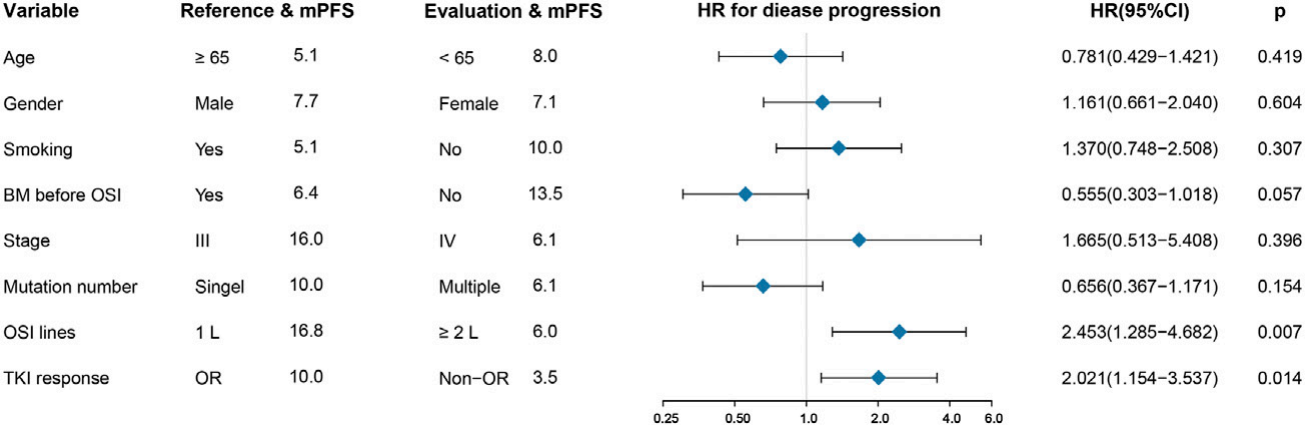
Univariate analysis for progression-free survival (PFS). First-line osimertinib and having an objective tumor response are associated with long PFS. BM: brain metastases; OSI: osimertinib.

All patients in the group had a median PFS of 7.1 months. Patients who underwent first-line treatment of osimertinib had an mPFS of 16.8 months, which was substantially higher than the mPFS of 6.0 months for those who got second- or later-line therapy (HR: 2.453, 95%CI: 1.285–4.682, *p* =0.004) (Figure 2, Supplementary Figure S3C). In addition, patients who presented an objective response to osimertinib had a considerably superior PFS than those who did not, with mPFS values of 10.0 and 3.5 months, respectively. (HR: 2.021, 95%CI: 1.154–3.537, *p* =0.014) (Figure 2, Supplementary Figure S3D). However, in terms of brain metastasis status, although the mPFS in patients without brain metastases before treatment with osimertinib was more than twice as high as that in those suffering from brain metastases (13.5 months versus 6.4 months) and the Kaplan–Meier curves also showed a trend favoring patients without brain metastases, however, there was no statistically significant difference between them. (*p* = 0.055) (Supplementary Figure S3A). In addition, there was no significant difference in PFS between patients with single or multiple ucm-EGFRms (Supplementary Figure S3B).

### 3.4 Exploratory analysis

**FIGURE 3:**
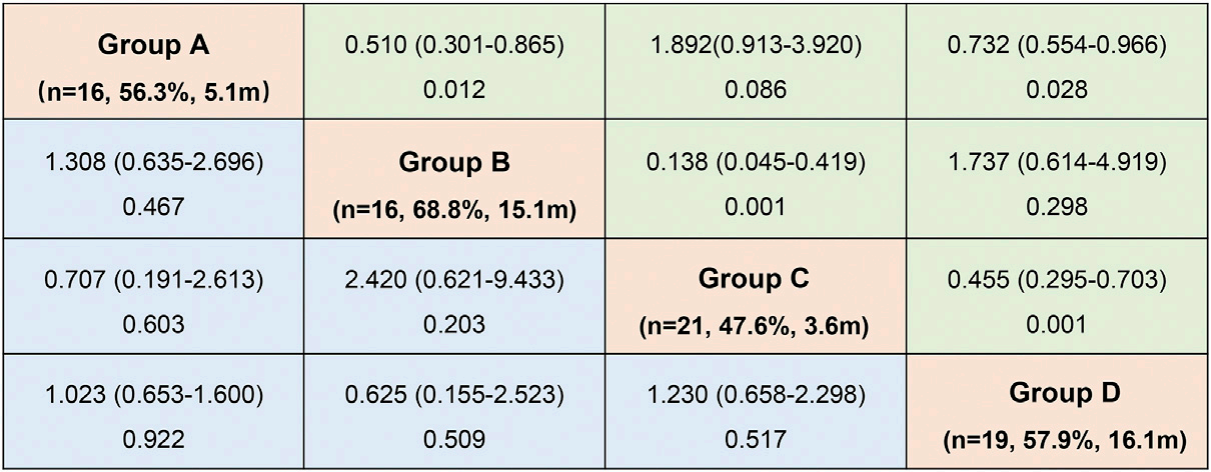
Odds ratio (ORa)with 95% CI for objective response rate (ORR) (blue)and hazard ratio (HR)with 95% CI for progression-free survival (PFS) (green) in subgroup analysis according to mutation patterns (OR and HR was set by column versus row). (Group **(A)**: exon 20 insertions; Group **(B)**: other single ucm-EGFRms; Group **(C)**: multiple ucm-EGFRms that with T790M; Group **(D)**: multiple ucm-EGFRms that without T790M).

**FIGURE 4:**
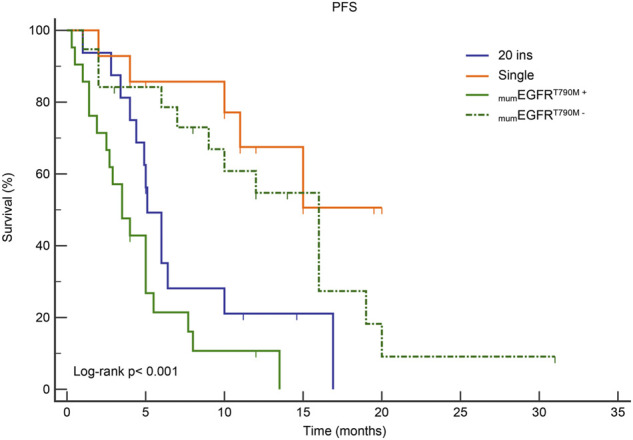
Kaplan-Meier curves for PFS in subgroup analysis according to mutation patterns. _mum_EGFR^T790M+^: multiple ucm-EGFRms that with T790M; _mum_EGFR^T790M−^: multiple ucm-EGFRms that without T790M.

**FIGURE 5:**
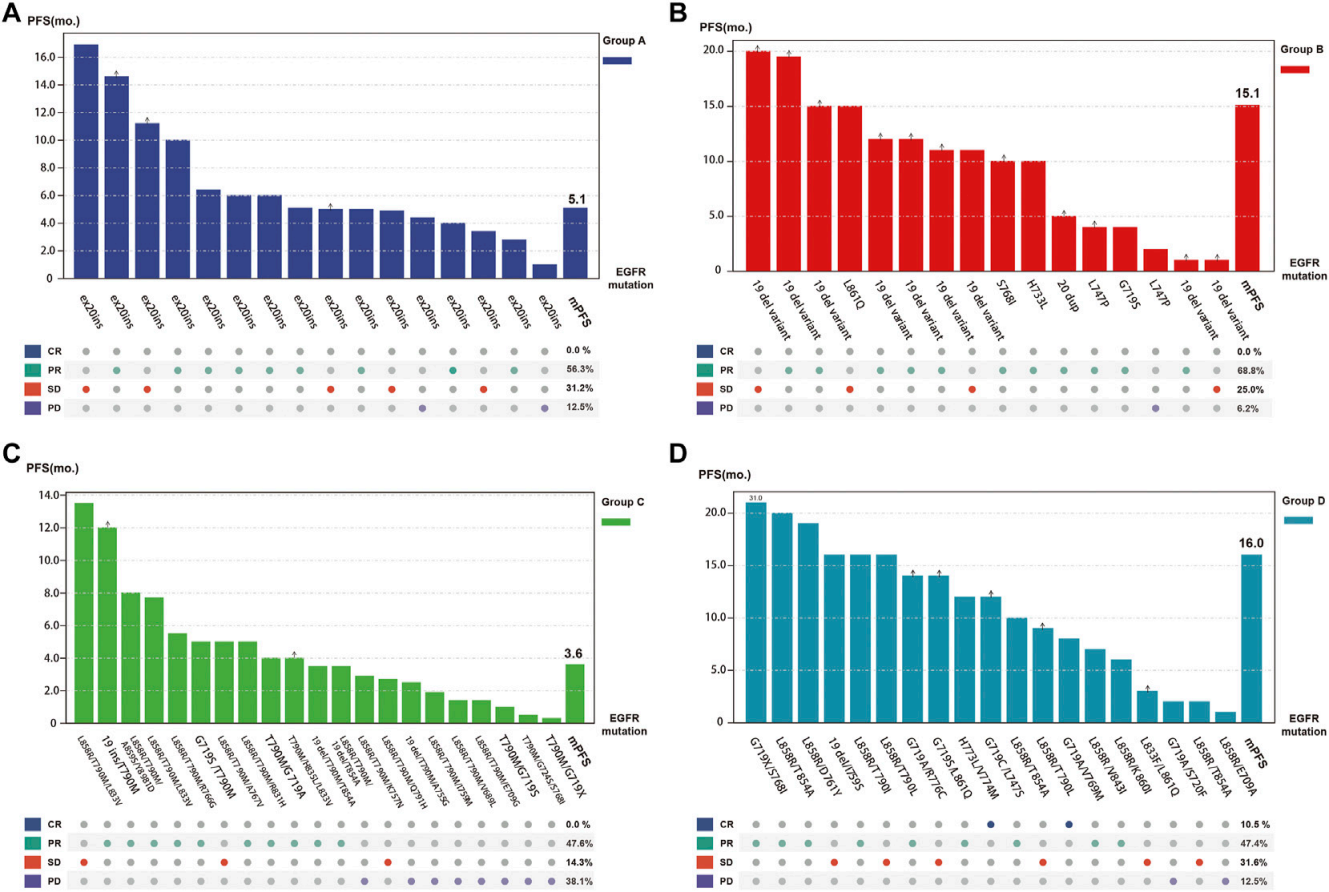
Tumor response and progression-free survival (PFS) of each individual patient as well as overall tumor objective response rate and median PFS for each subgroup (Group **(A)**, exon 20 insertions; Group **(B)**, other single ucm-EGFRms; Group **(C)**, multiple ucm-EGFRms that with T790M; Group **(D)**, multiple ucm-EGFRms that without T790M).

Patients in Group B had the best response to osimertinib, with an ORR of 68.8% and a median PFS of 15.1 months, according to subgroup analysis. Followed by group D, with an ORR of 57.9% and an mPFS of 16.1 months. In contrast, the efficacy of osimertinib and prognoses was worst for patients in Group C, with an ORR of 47.6% and an mPFS of only 3.6 months, respectively (Figure 3 and Figure 4). Although the ORR varied between the groups (47.6%–68.8%), when a one-by-one comparison was conducted, the difference was not statistically significant (all *p* > 0.05) (Figure 5). Regarding the PFS, Group B showed a significantly higher PFS than Groups A and C (*p* =0.012, *p* <0.001, respectively), but the difference from that of Group D was not statistically significant (*p* =0.298). Additionally, PFS differences between Group C with Groups B (*p* <0.001) and D (*p* <0.001) were statistically significant. However, when compared with Group A, the differences were not statistically significant, although a tendency in favor of group A was revealed (*p* =0.086) (Figure 5). The PFS of each patient and the efficacy of osimertinib in different groups are shown in Figure 3.

## 4 Discussion

The present study showed the largest sample size and most comprehensive analysis of clinical outcomes of osimertinib-treated NSCLC patients with ucm-EGFRms. The ORR was 57.0%, and the mPFS was 7.1 months after receiving osimertinib. The ORR and mPFS following first-line osimertinib treatment were both superior to those of patients receiving second- or later-line therapies, with the ORR and mPFS being 67.9% and 16.8 months, respectively. We also reported better response rates as well as an encouraging progression-free survival benefit in those who harbor a non-ex20ins, single ucm-EGFRm. In addition, patients carrying multiple ucm-EGFRms with T790 M simultaneously exhibited lower sensitivity to osimertinib.

The clinicopathological characteristics of uncommon and common EGFR mutations have been demonstrated to be similar, in comparison to common EGFR mutations, however, ucm-EGFRms are less sensitive and responsive to EGFR-TKI therapy (Russo et al., 2019; John et al., 2022). Patients with NSCLC harboring ucm-EGFRm had a poorer response, lower ORR, and shorter PFS than patients with 19del/L858R after receiving EGFR-TKIs (Wu et al., 2011; Watanabe et al., 2014; Yang et al., 2015; Yang et al., 2020). The ORR was 47.5%, with an mPFS at 5.0 months, according to a retrospective analysis of 61 patients with ucm-EGFRms who received first-generation EGFR-TKIs (Wu et al., 2011). Moreover, a post hoc examination of the NEJ002 clinical studies revealed that the ORR in patients with ucm-EGFRms who received gefitinib was only 20.0%, with an mPFS of 2.20 months (Watanabe et al., 2014). For patients treated with afatinib, a post-hoc examination of the data from the LUX-Lung 2, 3, and six clinical studies revealed that patients with ucm-EGFRms other than T790M and ex20ins following first-line afatinib had an ORR of 71.0%, with an mPFS at 10.7 months (95% CI: 5.6–14.7 months) following first-line afatinib (Yang et al., 2015). Another study evaluated the clinical therapeutic effectiveness of afatinib in 315 patients who carried ucm-EGFRms in randomized clinical trials or real-world cases. The results showed that patients treated with afatinib who harbored major ucm-EGFRms and harbored multiple ucm-EGFRms had ORRs of 60.0% and 77.1%, accordingly, with corresponding median times to treatment failure were 10.8 months and 14.7 months (Yang et al., 2020). Recently, a phase II clinical trial with a small sample of NSCLC patients harboring ucm-EGFRms indicated that osimertinib exhibited clinical activity against ucm-EGFRms. There are 36 patients who received the treatment of osimertinib and presented an ORR of 50.0% and a corresponding mPFS of 8.2 months (Cho et al., 2020). Our findings were similar to those of prior research in which 72 patients were treated with osimertinib, with ORR and mPFS of 57.0 percent and 7.1 months. In addition, a retrospective study reported that patients harboring ucm-EGFRms who received osimertinib for first-line treatment had a median time on osimertinib of 8.9 months (95% CI, 7.0–10.8 months), superior to that of the overall cohort (7.1 m, 95% CI, 5.4–8.8 months) (Ji et al., 2020). In the current research, osimertinib was used as first-line treatment in 28 patients, with an ORR of 67.9% and a median PFS of 16.8 months, both superior to those of patients treated in second- or later-line therapy as well as those of the overall cohort. Nevertheless, due to the low mutation frequency as well as high heterogeneity of this category of mutations, currently, available clinical data on the efficacy of osimertinib are insufficient and require further elucidation and validation. Notably, there are several ongoing phase II clinical trials aimed at evaluating the efficiency of osimertinib against ucm-EGFRms (NCT03434418; NCT03191149; NCT13414814).

Data from clinical trials and real-world research reveal that patients with ucm-EGFRms exhibit inconsistent responses and survival outcomes following EGFR-TKI treatment that are closely related to the mutation pattern and the cooccurring partner mutant genes (Russo et al., 2019; John et al., 2022). Therefore, we conducted a subgroup analysis to look into the differences in osimertinib efficacy and the resulting prognoses of patients with different ucm-EGFRm types to identify which, if any, potential subcohort of patients who are more likelihood to experience gains from osimertinib. Ex20ins are the third most frequent EGFR mutation, representing about 10–12% of all EGFR mutations (Russo et al., 2019). However, due to its induction of steric hindrance at the drug-binding pocket, most of the EGFR proteins harboring these mutations are relatively insensitive to first- or second-generation EGFR-TKIs (Russo et al., 2019; John et al., 2022). A retrospective study including 21 patients with ex20ins reported an ORR of 5.0% and an mPFS of 3.6 months (95% CI: 2.6–4.5 mo), respectively, after patients received osimertinib (20 of 80 mg/day and one of 160 mg/day) (van Veggel et al., 2020). A phase II clinical study (NCT03414814) reported similar survival data: the mPFS was 3.5 months in patients with ex20ins who received osimertinib (Kim et al., 2019). In the present research, 16 patients carrying ex20ins who received osimertinib presented an ORR of 56.3% and an mPFS of 5.1 months, which are higher than the values reported above. Surprisingly, a recent small sample phase II trial (ECOG-ACRIN 5162) showed that a high dose of osimertinib of 160 mg daily yielded better outcomes in patients harboring ex20ins, with an ORR of 25.0%, an mPFS at 9.7 months (Piotrowska et al., 2020). Another phase II trial (POSITION20) demonstrated the effectiveness of applying 160 mg daily of osimertinib in patients harboring ex20ins with an ORR of 27.0% and mPFS of 5.5 months (Zwierenga et al., 2021). The above data may indicate that high doses of osimertinib may overcome the intrinsic resistance of ex20ins. Nevertheless, considering the limited sample size and the great variability of ex20ins, the clinical benefit of this approach for this group of patients requires further elucidation. Encouragingly, in contrast to ex20ins, osimertinib showed promising efficacy in patients with other types of single ucm-EGFRmuts in this study, with an ORR of 68.8% and mPFS of 15.1 months. Previous studies have demonstrated the efficacy of osimertinib in patients with several certain types of ucm-EGFRms. For example, in the KCSG-LU15-09 trial, patients carrying G719X, S768I, and L861Q had ORRs of 53.0%, 78.0%, and 38.0% after osimertinib treatment, respectively, with corresponding median PFS times of 8.2, 15.2, and 12.3 months, respectively, which represents the best-demonstrated efficacy of osimertinib in patients with single, non-ex20ins, ucm-EGFRms (Cho et al., 2020). Consistently, a multicenter retrospective study also reported favorable efficacy of osimertinib in patients carrying the L861Q and G719X mutations, with median durations on first-line osimertinib at 19.3 months and 5.8 months, respectively (Ji et al., 2020). Moreover, preclinical studies and clinical data from real-world cases or case series have also confirmed the effectiveness of osimertinib against G719X, L861Q, and S768I (Floc'h et al., 2020; Bar et al., 2021). Therefore, it might be considered an effective treatment option to apply osimertinib to treat patients with single, non-ex20ins, ucm-EGFRms, particularly those with G719X, L861Q, and S768I, in some circumstances.

T790M mutation is the primary mechanism of drug resistance for first- or second-generation EGFR-TKIs (Westover et al., 2018). Osimertinib is irreversible EGFR-TKI that demonstrated selective inhibition of both the sensitive mutation 19del/L858R as well as the T790 M resistance mutation (Cross et al., 2014). It is currently approved for the treatment of T790M-mutated advanced NSCLC patients who experienced disease progression during the treatment of first-line EGFR-TKIs (Mok et al., 2017). However, there are limited data on the effectiveness of osimertinib in the treatment of T790M-positive patients with ucm-EGFRms. In this research, 21 patients carried multiple ucm-EGFRms that contained T790M, with an ORR of 47.6% and an mPFS of 3.6 months, the lowest values observed for all subgroups. In contrast, patients with multiple ucm-EGFRms without T790M showed favorable outcomes, with an ORR of 60.0% and an mPFS of 12.1 months, which is even comparable to the outcomes in the Phase III AURA clinical trial (Mok et al., 2017). These findings are consistent with previous studies in which osimertinib demonstrated inferior efficacy in patients with multiple ucm-EGFRms that contained T790M. The UNICORN case series reported nine patients with the T790M mutation (5 with 19del/L858R) treated with osimertinib, with an ORR of 33.3%, which was lower than that in the overall cohort (Bar et al., 2021). Moreover, Si et al. reported an even lower ORR of only 10% in 11 patients carrying the T790M mutation who were treated with osimertinib (Si et al., 2021). Additionally, the real-world study ASTRIS (NCT02474355) also concluded that patients with ucm-EGFRms containing T790M had a poorer response to osimertinib, with a lower ORR and shorter PFS (Cheema et al., 2019). In brief, for patients with multiple ucm-EGFRms that contain T790M, given the wide heterogeneity of ucm-EGFRms and the limited clinical data available, clinicians should make prudent clinical decisions based on complete comprehension of the sensitivity and resistance of known mutated genes, especially concurrent partner mutations.

Brain metastasis (BM) is a common occurrence in advanced NSCLC patients and has a massive effect on their prognosis and quality of life. Preclinical investigations have demonstrated that osimertinib has a stronger blood-brain barrier penetration capability than first- and second-generation EGFR-TKIs (Ballard et al., 2016). This finding was also demonstrated by clinical trials where osimertinib showed favorable intracranial efficacy and significant improvement in central nervous system (CNS) remission rates (Reungwetwattana et al., 2018; Ahn et al., 2020). The correlation between pretreatment BM status and osimertinib efficacy was investigated in this study. The data demonstrated a trend in favor of patients without BMs, but the difference was not statistically significant, despite the fact that patients without BMs had an mPFS that was more than twice that of patients with BMs. This result is in line with the findings of phase II clinical research (KCSG-LU15-09) indicating that patients with central nervous system metastases had a shorter mPFS than those without (5.4 months versus 9.8 months); in addition, in the five patients with evaluable central nervous system response, the intracranial ORR was 40% (Cho et al., 2020). This finding may indicate that osimertinib alone may have limited efficacy in treating brain metastasis in patients with ucm-EGFRms.

This research has several inevitable limitations. To begin with, this is a re-analysis based on published research. This may be affected by, such as selection bias, publication bias, and other uncontrollable confounding factors. Secondly, limited by the sample size, the efficacy of osimertinib alone in the treatment of brain metastases in patients with ucm-EGFRms remains to be further validated in clinical practice. In addition, due to the variability of the included articles, there were not enough data for comparison of drug toxicity and side effects. Therefore, further large-scale, randomized controlled clinical research is necessary to validate the findings.

## 5 Conclusion

In summary, osimertinib treatment exhibits favorable but inconsistent efficacy in patients with ucm-EGFRms, which is closely related to the mutation pattern and the cooccurring partner mutant genes. NSCLC patients carrying ucm-EGFRms could be classified into various sub-cohorts that displayed different responses and survival outcomes following osimertinib treatment, which requires further clinical studies for verification.

## Data Availability

The raw data supporting the conclusions of this article will be made available by the authors, without undue reservation.

## Supplementary material

Supplementary Figure S1 Flow chart of the study process. Overall, forty-four articles were selected for inclusion in this pooled analysis.

Supplementary Figure S2 The composition of uncommon EGFR mutations (*n*=72). The number and percentage of the top 6 uncommon EGFR mutations are shown, and mutations with lower frequencies are listed below, each of which has only one patient, accounting for 1.4%.

Supplementary Figure S3 Kaplan-Meier curves for progression-free survival (PFS) according to brain metastases (BM) status before OSI **(A)**, mutation number **(B)**, OSI-lines **(C)**, and treatment response **(D)**. BM: brain metastases; OSI: osimertinib.
