# Supplementary figures and images for: Insensitivity to T790M mutation? A pooled analysis of outcomes following osimertinib for the treatment of NSCLC patients harboring uncommon epidermal growth factor receptor mutation
Source: Front Pharmacol. 2022 Aug 26;13:986962. doi: 10.3389/fphar.2022.986962 (PMC9458881; doi:10.3389/fphar.2022.986962)

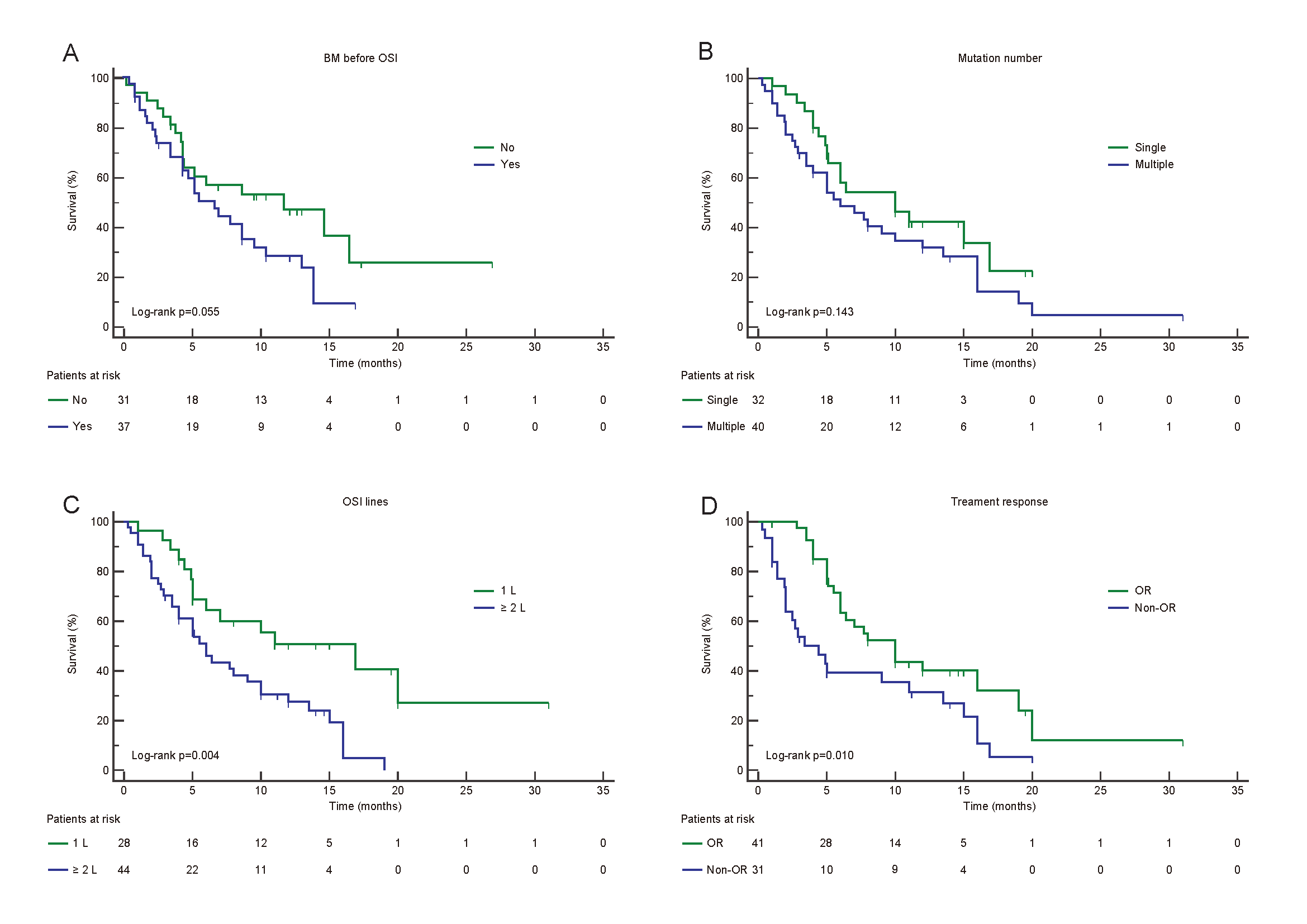

Supplement: Supplementary file 1 [file Image3.TIF]

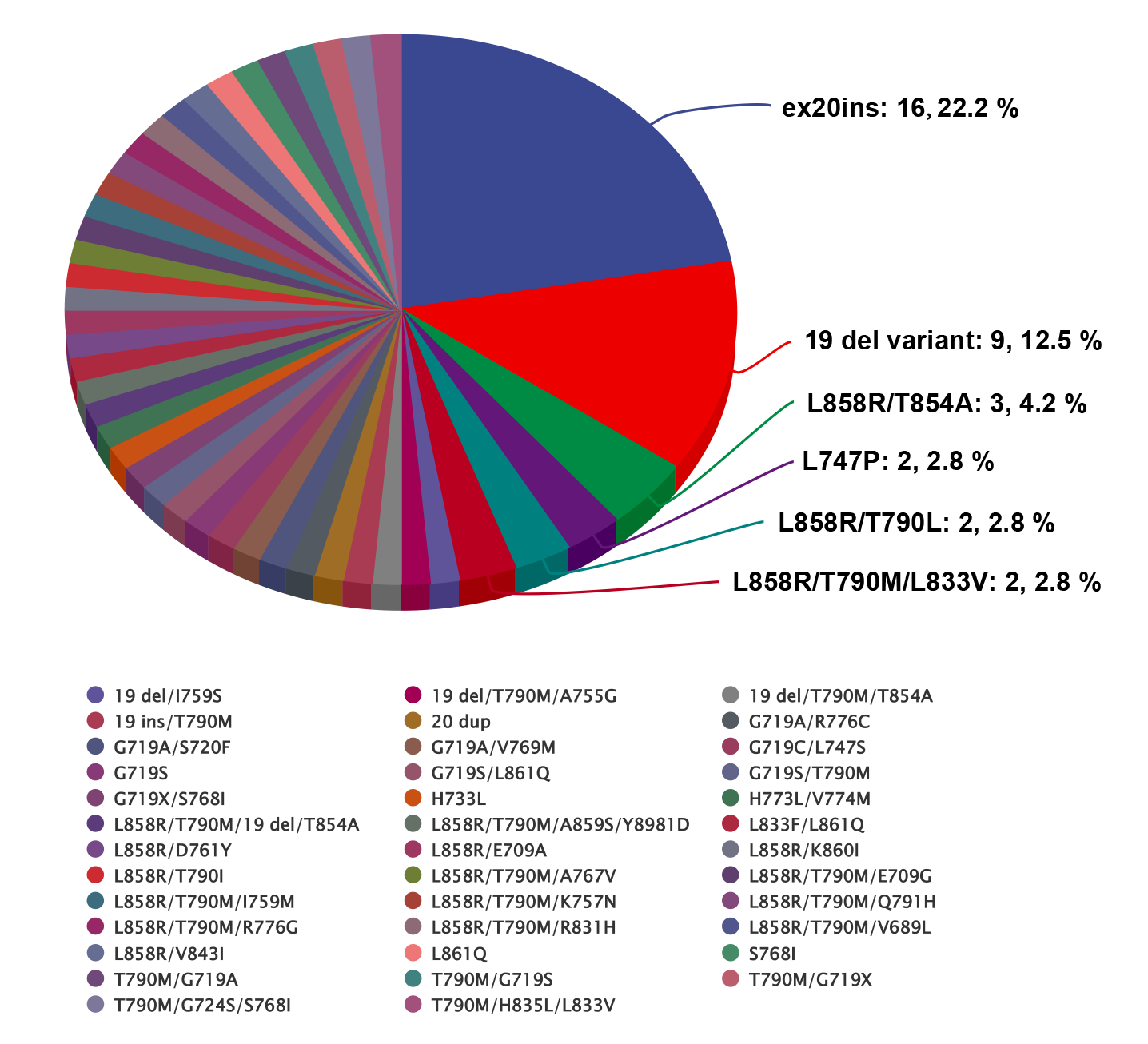

Supplement: Supplementary file 2 [file Image2.TIF]

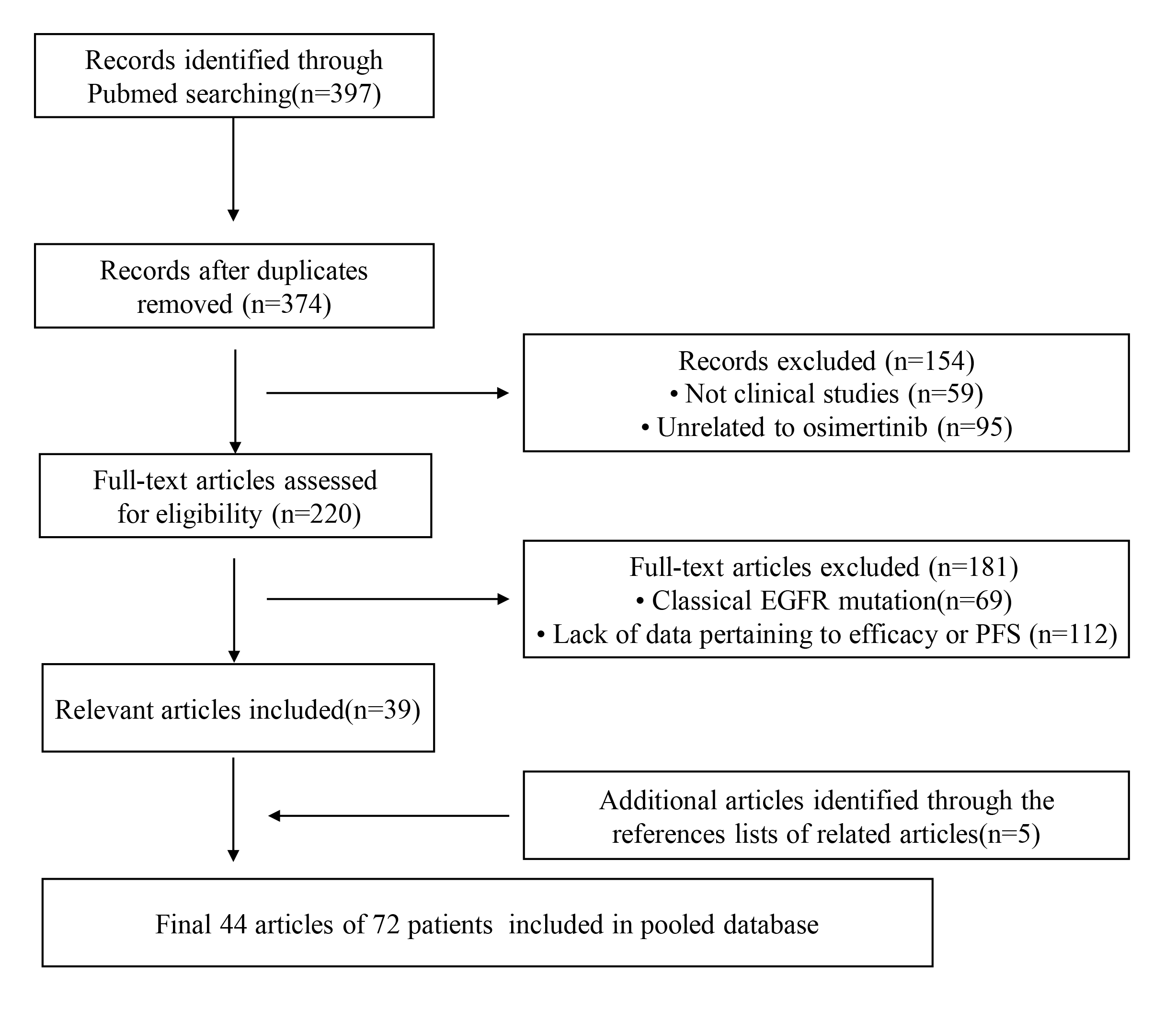

Supplement: Supplementary file 3 [file Image1.TIF]
