# Supplementary material for: Insensitivity to T790M mutation? A pooled analysis of outcomes following osimertinib for the treatment of NSCLC patients harboring uncommon epidermal growth factor receptor mutation
Source: Front Pharmacol. 2022 Aug 26;13:986962. doi: 10.3389/fphar.2022.986962 (PMC9458881; doi:10.3389/fphar.2022.986962)
Supplement: Supplementary file 4 [file DataSheet2.DOCX]

Supplement Table 1. The studies included for this pooled analysis

| Study | Study type | years | No. of patients | References* |
| --- | --- | --- | --- | --- |
| Chinese retrospective study | Single-center China study in patients with complex EGFR mutations | 2020 | 11 | [1] |
| Chinese retrospective study | Single-center China study in patients with exon 20 insertion mutations treated with osiertinib | 2019 | 6 | [2] |
| Chinese retrospective study | Single-center China study in patients with EGFR T854A mutations treated with osimertinib | 2021 | 5 | [3] |
| India retrospective study | Single-center India study in patients with rare EGFR mutations | 2021 | 2 | [4] |
| Chinese retrospective study | Single-Center China study in patients with exon 19 insertion-deletion variants | 2020 | 8 | [5] |
| Case reports/case series | Various(n=39) | 2018-2022 | 40 | [6-44] |

*: References here refer to the articles included in this pooled-analysis, not the references cited in the manuscript, which are listed in the reference section of the manuscript.

**References**

1. Lin YT, Tsai TH, Wu SG, Liu YN, Yu CJ, Shih JY. Complex EGFR mutations with secondary T790M mutation confer shorter osimertinib progression-free survival and overall survival in advanced non-small cell lung cancer. Lung Cancer. 2020 Jul;145:1-9.

2. Fang W, Huang Y, Hong S, Zhang Z, Wang M, Gan J, et al. EGFR exon 20 insertion mutations and response to osimertinib in non-small-cell lung cancer. BMC Cancer. 2019 Jun 17;19(1):595.

3. Zhang L, Yang X, Ming Z, Shi J, Lv X, Li W, et al. Molecular Characteristics of the Uncommon EGFR Exon 21 T854A Mutation and Response to Osimertinib in Patients With Non-Small Cell Lung Cancer. Clin Lung Cancer. 2021 Dec 24.

4. Mehta A, Vasudevan S. Rare epidermal growth factor receptor gene alterations in non-small cell lung cancer patients, tyrosine kinase inhibitor response and outcome analysis. Cancer Treat Res Commun. 2021 May 13;28:100398.

5. Peng X, Long X, Liu L, Zeng L, Yang H, Jiang W, et al. Clinical impact of uncommon epidermal growth factor receptor exon 19 insertion-deletion variants on epidermal growth factor receptor-tyrosine kinase inhibitor efficacy in non-small-cell lung cancer. Eur J Cancer. 2020 Dec;141:199-208.

6. Huang J, Wang Y, Zhai Y, Wang J. Non-small cell lung cancer harboring a rare EGFR L747P mutation showing intrinsic resistance to both gefitinib and osimertinib (AZD9291): A case report. Thorac Cancer. 2018 Jun;9(6):745-49.

7. Nasu S, Shiroyama T, Morita S, Takata S, Takada H, Masuhiro K, et al. Osimertinib Treatment Was Unsuccessful for Lung Adenocarcinoma with G719S, S768I, and T790M Mutations. Intern Med. 2018 Dec 15;57(24):3643-45.

8. Piotrowska Z, Fintelmann FJ, Sequist LV, Jahagirdar B. Response to Osimertinib in an EGFR Exon 20 Insertion-Positive Lung Adenocarcinoma. J Thorac Oncol. 2018 Oct;13(10):e204-e06.

9. Qin BD, Jiao XD, Yuan LY, Liu K, Wang Z, Qin WX, et al. The effectiveness of afatinib and osimertinib in a Chinese patient with advanced lung adenocarcinoma harboring a rare triple EGFR mutation (R670W/H835L/L833V): a case report and literature review. Onco Targets Ther. 2018;11:4739-45.

10. Yang M, Tong X, Xu X, Zheng E, Ni J, Li J, et al. Case Report: Osimertinib achieved remarkable and sustained disease control in an advanced non-small-cell lung cancer harboring EGFR H773L/V774M mutation complex. Lung Cancer. 2018 Jul;121:1-4.

11. Byeon S, Kim Y, Lim SW, Cho JH, Park S, Lee J, et al. Clinical Outcomes of EGFR Exon 20 Insertion Mutations in Advanced Non-small Cell Lung Cancer in Korea. Cancer Res Treat. 2019 Apr;51(2):623-31.

12. Cai Y, Wang X, Guo Y, Sun C, Xu Y, Qiu S, et al. Successful treatment of a lung adenocarcinoma patient with a novel EGFR exon 20-ins mutation with afatinib: A case report. Medicine (Baltimore). 2019 Jan;98(1):e13890.

13. Fang W, Huang Y, Gan J, Hong S, Zhang L. A Patient with EGFR Exon 20 Insertion-Mutant Non-Small Cell Lung Cancer Responded to Osimertinib plus Cetuximab Combination Therapy. J Thorac Oncol. 2019 Sep;14(9):e201-e02.

14. Grolleau E, Haddad V, Boissiere L, Falchero L, Arpin D. Clinical Efficacy of Osimertinib in a Patient Presenting a Double EGFR L747S and G719C Mutation. J Thorac Oncol. 2019 Jul;14(7):e151-e53.

15. Hakozaki T, Yomota M. Acquisition of T790M resistance mutation in a patient with advanced adenocarcinoma harbouring uncommon EGFR mutations: a case report and literature review. Onco Targets Ther. 2019;12:745-48.

16. Ikari T, Sakakibara-Konishi J, Yamamoto G, Kitai H, Mizugaki H, Asahina H, et al. Response to First-Line Osimertinib Treatment in Non-Small-Cell Lung Cancer With Coexisting G719A and Primary T790M Epidermal Growth Factor Receptor Mutations. Clin Lung Cancer. 2019 Jul;20(4):e531-e33.

17. Murano C, Igarashi A, Yamauchi K, Inoue S, Watanabe M. Osimertinib as treatment for EGFR exon 20 insertion-positive lung adenocarcinoma. EXCLI J. 2019;18:893-98.

18. Cai Y, Wang Y, Sun J, Wang X, Xu Y, Sun C, et al. Successful treatment of a patient with NSCLC carrying uncommon compound EGFR G719X and S768I mutations using osimertinib: A case report. J Int Med Res. 2020 Jun;48(6):300060520928793.

19. Coleman N, Woolf D, Welsh L, McDonald F, MacMahon S, Yousaf N, et al. EGFR Exon 20 Insertion (A763_Y764insFQEA) Mutant NSCLC Is Not Identified by Roche Cobas Version 2 Tissue Testing but Has Durable Intracranial and Extracranial Response to Osimertinib. J Thorac Oncol. 2020 Oct;15(10):e162-e65.

20. Huang X, Yang Y, Wang P, Wang J, Chen S, Mao X, et al. A rare EGFR mutation L747P conferred therapeutic efficacy to both ge fi tinib and osimertinib: A case report. Lung Cancer. 2020 Dec;150:9-11.

21. Inagaki Y, Tamiya A, Matsuda Y, Azuma K, Adachi Y, Enomoto T, et al. Poor effect of osimertinib on EGFR exon 20 insertion-positive lung adenocarcinoma: A case report. Medicine (Baltimore). 2020 Oct 16;99(42):e22628.

22. Li H, Yu T, Lin Y, Xie Y, Feng J, Huang M, et al. Three Novel EGFR Mutations (750_758del, I759S, T751_I759delinsS) in One Patient with Metastatic Non-Small Cell Lung Cancer Responding to Osimertinib: A Case Report. Onco Targets Ther. 2020;13:7941-48.

23. Okuno T, Arakawa S, Yoshida T, Ohe Y. Efficacy of osimertinib in a patient with leptomeningeal metastasis and EGFR uncommon S768I mutation. Lung Cancer. 2020 May;143:95-96.

24. Onozawa H, Saito H, Sunami K, Kubo T, Yamamoto N, Kasajima R, et al. Lung adenocarcinoma in a patient with a cis EGFR L858R-K860I doublet mutation identified using NGS-based profiling test: Negative diagnosis on initial companion test and successful treatment with osimertinib. Thorac Cancer. 2020 Dec;11(12):3599-604.

25. Simionato F, Calvetti L, Cosci M, Scarparo S, Aprile G. Case Report: A Metabolic Complete Response to Upfront Osimertinib in a Smoker Non-Small Cell Lung Cancer Patient Harbouring EGFR G719A/V769M Complex Mutation. Onco Targets Ther. 2020;13:12027-31.

26. Song H, Chen Y, Yan Z, Sun G, Shi Z. Response to Osimertinib in a NSCLC Patient Harboring EGFR V843I Germ-Line Mutation. Lung Cancer. 2020 Dec;150:247-48.

27. Vasconcelos P, Gergis C, Viray H, Varkaris A, Fujii M, Rangachari D, et al. EGFR-A763_Y764insFQEA Is a Unique Exon 20 Insertion Mutation That Displays Sensitivity to Approved and In-Development Lung Cancer EGFR Tyrosine Kinase Inhibitors. JTO Clin Res Rep. 2020 Sep;1(3).

28. Yang Y, Zhang X, Wang R, Qin J, Wang J, Li Z, et al. Osimertinib Resistance With a Novel EGFR L858R/A859S/Y891D Triple Mutation in a Patient With Non-Small Cell Lung Cancer: A Case Report. Front Oncol. 2020;10:542277.

29. Zhang C, Lin L, Zuo R, Wang Y, Chen P. Response to tyrosine kinase inhibitors in lung adenocarcinoma with the rare epidermal growth factor receptor mutation S768I and G724S: A case report and literature review. Thorac Cancer. 2020 Sep;11(9):2743-48.

30. Zhang Q, Jiang T, Xiao M, Zhang J, Ying X. Identification of a Novel Osimertinib-Sensitive Mutation, EGFR H773L, in a Chinese Patient With NSCLC. J Thorac Oncol. 2020 Mar;15(3):e46-e48.

31. Zhu Y, Tang J, Li X, Qin T, Wei Y. Durable Response to Osimertinib in a Chinese Patient with Metastatic Lung Adenocarcinoma Harboring a Rare EGFR L858R/D761Y Compound Mutation. Onco Targets Ther. 2020;13:10447-51.

32. Fang YF, Liu PC. Afatinib and osimertinib in lung adenocarcinoma harbored EGFR T751_I759delinsS mutation: A case report. Thorac Cancer. 2021 Dec;12(24):3429-32.

33. Huang X, Yang Y, Wang P, Han-Zhang H, Ding L. A heavily pre-treated adenocarcinoma patient with EGFR exon 20 insertion mutation responded to pembrolizumab plus nab-paclitaxel/bevacizumab: a case report. Ann Palliat Med. 2021 Jun;10(6):6997-7002.

34. Kunimasa K, Nishino K, Kukita Y, Matsumoto S, Kawachi H, Kawamura T, et al. Late recurrence of lung adenocarcinoma harboring EGFR exon 20 insertion (A763_Y764insFQEA) mutation successfully treated with osimertinib. Cancer Genet. 2021 Aug;256-257:57-61.

35. Lin R, Chen R, Chen Z, Hu L, Guo W, Zhang Z, et al. Efficacy of Osimertinib in NSCLC Harboring Uncommon EGFR L861Q and Concurrent Mutations: Case Report and Literature Review. Front Oncol. 2021;11:731572.

36. Sehgal K, Rangachari D, VanderLaan PA, Kobayashi SS, Costa DB. Clinical Benefit of Tyrosine Kinase Inhibitors in Advanced Lung Cancer with EGFR-G719A and Other Uncommon EGFR Mutations. Oncologist. 2021 Apr;26(4):281-87.

37. Shan CG, Wang H, Lin T, Liu D, Wen L, Chen ZJ, et al. A non-small cell lung cancer (NSCLC) patient with leptomeningeal metastasis harboring rare epidermal growth factor receptor (EGFR) mutations G719S and L861Q benefited from doubling dosage of osimertinib: a case report. Ann Palliat Med. 2021 May;10(5):5897-901.

38. Tamiya M, Kunimasa K, Nishino K, Matsumoto S, Kawachi H, Kuno K, et al. Successful treatment of an osimertinib-resistant lung adenocarcinoma with an exon 18 EGFR mutation (G719S) with afatinib plus bevacizumab. Invest New Drugs. 2021 Feb;39(1):232-36.

39. Wang Y, Liu S, Feng A, Luo H, Hu J, Wang K, et al. Identification of a Rare EGFR T790I Mutation in Lung Adenocarcinoma Sensitive to Osimertinib. Front Oncol. 2021;11:727312.

40. Xiang C, Zhang W, Xiong LW, Cai XW, Teng HH, Zhao RY, et al. EGFR Thr790Leu as a Potential Resistance Mechanism to First-Generation EGFR Tyrosine Kinase Inhibitor May Respond to Osimertinib in Patients With Lung Adenocarcinoma. JTO Clin Res Rep. 2021 Jul;2(7):100185.

41. Zhang Y, Shen JQ, Shao L, Chen Y, Lei L, Wang JL. Non-small-cell lung cancer with epidermal growth factor receptor L861Q-L833F compound mutation benefits from both afatinib and osimertinib: A case report. World J Clin Cases. 2021 Sep 26;9(27):8220-25.

42. Zhao Y, Zhai L, Deng L, Halmos B, Cheng H. Efficacy of Osimertinib in Afatinib-resistant Lung Cancer Harboring Uncommon EGFR Mutations: Case Report and Literature Review. Clin Lung Cancer. 2021 May;22(3):e466-e69.

43. Zhi X, Luo J, Li W, Wang J, Wang Y, Cai Y, et al. Case Report: Osimertinib Followed by Osimertinib Plus Bevacizumab, Personalized Treatment Strategy for a Lung Cancer Patient With a Novel EGFR Exon 20 Insertion D770_N771insGT and Multiple Brain Metastases. Front Oncol. 2021;11:733276.

44. Ito N, Masuda T, Ooka I, Hosoya T, Yamaguchi K, Sakamoto S, et al. First-line osimertinib treatment in a patient with lung adenocarcinoma with coexisting epidermal growth factor receptor G719S and de novo T790M mutations. Thorac Cancer. 2022 Jan 25.
