# Supplementary material for: Insensitivity to T790M mutation? A pooled analysis of outcomes following osimertinib for the treatment of NSCLC patients harboring uncommon epidermal growth factor receptor mutation
Source: Front Pharmacol. 2022 Aug 26;13:986962. doi: 10.3389/fphar.2022.986962 (PMC9458881; doi:10.3389/fphar.2022.986962)
Supplement: Supplementary file 5 [file DataSheet1.DOCX]

The following search strategy were used: Search (((((uncommon[Title/Abstract]) OR rare[Title/Abstract])) AND (("Mutation"[Mesh]) OR Mutations[Title/Abstract])) AND (("Receptor, Epidermal Growth Factor"[Mesh]) OR (((((Epidermal Growth Factor Receptor Kinase[Title/Abstract]) OR Epidermal Growth Factor Receptor Protein-Tyrosine Kinase[Title/Abstract]) OR Epidermal Growth Factor Receptor Protein Tyrosine Kinase[Title/Abstract]) OR EGF Receptor[Title/Abstract]) OR Epidermal Growth Factor Receptor[Title/Abstract]))) AND (((("Carcinoma, Non-Small-Cell Lung"[Mesh]) OR (((((((((Carcinomas, Non-Small-Cell Lung[Title/Abstract]) OR Lung Carcinoma, Non-Small-Cell[Title/Abstract]) OR Lung Carcinomas, Non-Small-Cell[Title/Abstract]) OR Non-Small-Cell Lung Carcinomas[Title/Abstract]) OR Nonsmall Cell Lung Cancer[Title/Abstract]) OR Non-Small-Cell Lung Carcinoma[Title/Abstract]) OR Non Small Cell Lung Carcinoma[Title/Abstract]) OR Carcinoma, Non-Small Cell Lung[Title/Abstract]) OR Non-Small Cell Lung Cancer[Title/Abstract]))) OR (("Lung Neoplasms"[Mesh]) OR (((((((((((((((((Pulmonary Neoplasms[Title/Abstract]) OR Neoplasms, Lung[Title/Abstract]) OR Lung Neoplasm[Title/Abstract]) OR Neoplasm, Lung[Title/Abstract]) OR Neoplasms, Pulmonary[Title/Abstract]) OR Neoplasm, Pulmonary[Title/Abstract]) OR Pulmonary Neoplasm[Title/Abstract]) OR Lung Cancer[Title/Abstract]) OR Cancer, Lung[Title/Abstract]) OR Cancers, Lung[Title/Abstract]) OR Lung Cancers[Title/Abstract]) OR Pulmonary Cancer[Title/Abstract]) OR Cancer, Pulmonary[Title]) OR Cancers, Pulmonary[Title/Abstract]) OR Pulmonary Cancers[Title/Abstract]) OR Cancer of the Lung[Title/Abstract]) OR Cancer of Lung[Title/Abstract]))).
